# Distinctive clinical phenotype of anti-centromere antibody-positive diffuse systemic sclerosis

**DOI:** 10.1093/rap/rky002

**Published:** 2018-03-07

**Authors:** Joana Caetano, Svetlana I Nihtyanova, Jennifer Harvey, Christopher P Denton, Voon H Ong

**Affiliations:** 1 Systemic Immunomediated Diseases Unit, Department of Medicine IV, Fernando Fonseca Hospital, Amadora, Portugal; 2 Centre for Rheumatology and Connective Tissue Diseases, University College London Medical School, Royal Free Hospital, London, UK; 3 Department of Clinical Immunology, Royal Free Hospital, London, UK

**Keywords:** systemic sclerosis, anti-centromere antibody, diffuse cutaneous subset, survival analysis

## Abstract

**Objectives:**

The aim was to define clinical characteristics and long-term survival of patients with dcSSc and positive ACA.

**Methods:**

We identified all cases of ACA^+^ SSc in our cohort (*n* = 1313). Those with dcSSc (ACA^+^ diffuse) were compared with representative groups of consecutive ACA^+^ patients with limited subset (ACA^+^ limited) and ACA^−^ dcSSc (non-ACA diffuse).

**Results:**

Thirty-five patients (2.7%) were ACA^+^ diffuse. The peak modified Rodnan skin score was not significantly different between the dcSSc subgroups, but it occurred later in the disease course in ACA^+^ diffuse (88.54 *vs* 30.65 months, *P* < 0.001). Patterns of organ involvement were different between the groups. ACA^+^ diffuse had a higher incidence of interstitial lung disease than ACA^+^ limited (22.86 *vs* 4.43%, *P* = 0.001), but lower than non-ACA diffuse (41.18%, *P* = 0.042). More patients developed pulmonary hypertension in the ACA^+^ diffuse group (28.5 *vs* 12.0% ACA^+^ limited or 12.0% non-ACA diffuse), although this was attributable to the longer follow-up in these patients. The cumulative incidence of pulmonary hypertension was not different from the other two groups. The incidence of cardiac involvement was similar between the dcSSc groups, and scleroderma renal crisis was more frequent in the non-ACA diffuse group. Survival in ACA^+^ patients was similar in both subsets, whereas non-ACA diffuse had higher mortality.

**Conclusion:**

ACA^+^ dcSSc is uncommon and has a distinct clinical phenotype, with a more insidious onset of skin and organ involvement. Even in dcSSc, ACA appears protective for organ-based complications, namely interstitial lung disease and scleroderma renal crisis, and is associated with a better survival than expected in dcSSc.

Key messages

- Although uncommon, ACA^+^ diffuse patients have a distinct clinical phenotype.
- ACA, or factors determining its development, may act as a phenotype modifier in diffuse SSc.
- Awareness of the trajectory of organ involvement in this subset may facilitate timely therapeutic interventions.

## Introduction

Although the pathogenic role of autoantibodies in scleroderma (SSc) is still unclear, there is strong evidence of a link between autoantibodies and organ complications and survival [1]. ACAs are the most frequent autoantibodies in SSc and are described as protective for scleroderma renal crisis (SRC) and interstitial lung disease (ILD) [2]. ACAs are typically associated with lcSSc, although a small proportion of ACA^+^ patients (5–7%), will have the diffuse cutaneous subset (dSSc) [2–4]. Both antibody specificity and disease subset may influence disease phenotypic expression and organ manifestations. Extensive skin involvement has been associated with more frequent internal organ involvement, mainly SRC and ILD, and with decreased survival in comparison with lcSSc [1, 5, 6].

The purpose of our study was to describe the clinical manifestations and long-term survival of ACA^+^ SSc patients with diffuse skin involvement (ACA^+^ diffuse), compared with two other subsets: ACA^+^ patients with lcSSc (ACA^+^ limited) and ACA^−^ with dcSSc (non-ACA diffuse). Our hypothesis is that ACA^+^ diffuse is a subgroup of SSc with distinct clinical manifestations.

## Materials and methods

### Study cohort

We identified all ACA^+^ SSc patients evaluated at the Centre for Rheumatology and Connective Tissue Diseases at the Royal Free Hospital between 2001 and 2015 (*n* = 1313). Of those, all consecutive ACA^+^ patients with dcSSc subset were selected (*n* = 35). Cutaneous involvement was defined as diffuse if skin thickening affected both distal and proximal areas to the elbows and knees, and as limited if skin thickening did not affect proximal areas. Comparative groups were defined as follows: 158 consecutive ACA^+^ limited and 258 consecutive non-ACA diffuse patients, from a well-characterized population of our SSc database. Patients without a fully established characterization were not included in the analysis as a comparison group. Comprehensive data were obtained from the Royal Free Hospital research database and integrated medical records review. All study patients fulfilled the 2013 ACR/EULAR classification criteria for SSc [7]. All procedures performed were in accordance with the ethical standards of the institutional research committee and with the 1964 Declaration of Helsinki and its later amendments, and informed consent was obtained from all study participants.

Autoantibodies were measured in an accredited institutional autoimmune serology laboratory using a validated in-house assay with appropriate quality control and blinded assessment of the results at time of reading. In brief, ANAs were identified by IIF on HEp-2 cell substrate, considered positive if titre ≥1/100; ACA and anti-U3-RNP were identified by indirect IIF on HEp-2 cell substrate; anti-Scl70, -nRNP, -Pm-Scl, -La and -Ro were identified by IIF and counter-immunoelectrophoresis; and anti-RNA polymerase III were identified by IIF and ELISA.

Clinical manifestations were recorded based on the assessment of the latest clinic visit. We used definitions of moderate-to-severe organ-based complications of SSc defined in previous studies [8]. SRC was defined as new onset of systemic hypertension >150/85 mmHg and a decrease in estimated glomerular filtration rate ≥30%, or SRC features in a renal biopsy. Pulmonary hypertension (PH) was defined as right heart catheterization with a mean pulmonary artery pressure of ≥25 mmHg and a normal pulmonary capillary wedge pressure. This included patients with CTD-associated pulmonary arterial hypertension and PH associated with ILD. ILD was confirmed by the presence of ground-glass opacities and/or honeycombing on high-resolution chest tomography, and clinically significant if forced vital capacity (FVC) or diffusing capacity for carbon monoxide (DLCO) ≤55% predicted or a documented decline in FVC or DLCO of ≥15%. Cardiac involvement was defined as haemodynamically significant cardiac arrhythmias, pericardial effusion or congestive heart failure requiring specific treatment in the absence of other known cardiac causes.

Disease onset was defined as the time since the first reported non-RP manifestation of SSc. Peak modified Rodnan skin score (mRSS) was defined as the highest mRSS recorded since disease onset and the latest clinic visit. Time to internal organ complications and time to death were defined as the time in months since SSc onset and the time point when the definition for significant organ involvement was fulfilled.

### Statistical analysis

Student’s *t*-test and Fisher’s exact test were used to compare demographic and clinical characteristics. Kaplan–Meier estimates of survival and 1 − Kaplan–Meier estimates of cumulative incidence of organ complications were calculated, and the log-rank test was used to compare those between the three groups. Analysis was carried out using STATA 14.

## Results

### Demographic and clinical characteristics

From a total of 1313 identified ACA^+^ SSc patients, 35 (2.7%) had the diffuse cutaneous subset. Table 1 shows the demographic, clinical characteristics and autoantibodies of the three groups.

**Table 1:** Demographic, serological and clinical characteristics of the three groups

	ACA^+^ diffuse,	ACA^+^ limited,	Non-ACA diffuse,	*P*-value
	*n* = 35	*n* = 158	*n* = 258	
Male, *n* (%)	6 (17.1)	14 (8.9)	55 (21.3)	0.003
Follow-up, mean (s.d.), months	172 (89)	124 (39)	104 (48)	<0.001
Age at disease onset, mean (s.d.), years	47 (11)	53 (13)	46 (13)	<0.001
Autoantibodies, *n* (%)				
Anti-Scl70	3 (8.6)	1 (0.6)	84 (32.6)	
Anti-RNA polymerase III	1 (2.9)	–	62 (24.0)	
Anti-nRNP	3 (8.6)	–	12 (4.6)	
Anti-U3-RNP	1 (2.9)	–	17 (6.6)	
Anti-Ro	1 (2.9)	3 (1.9)	9 (3.5)	
Anti-La	–	1 (0.6)	2 (0.8)	
Anti-Pm-Scl	–	–	14 (5.4)	
ANA negative	–	–	9 (3.5)	
ANA positive, ENA negative	–	–	54 (20.9)	
Peak mRSS, mean (s.d.)	24 (10)	7 (4)	27 (10)	<0.001
Time to peak mRSS, mean (s.d.), months	89 (78)	57 (43)	31 (33)	<0.001
Interstitial lung disease, *n* (%)	8 (22.9)	7 (4.4)	105 (41.9)	<0.001
Pulmonary hypertension, *n* (%)	10 (28.6)	19 (12.0)	31 (12.0)	0.036
Cardiac scleroderma, *n* (%)	3 (8.6)	3 (1.9)	16 (6.2)	0.052
Scleroderma renal crisis, *n* (%)	2 (5.7)	0 (0)	36 (13.9)	<0.001

The incidence of specific internal organ complications is based on the entire follow-up period. *P*-values are obtained from global comparison tests; ANOVA for the continuous variables and Fisher’s exact test for categorical variables. mRSS: modified Rodnan skin score.

At disease onset, patients with lcSSc were older than patients with dcSSc (*P* < 0.001). On average, peak mRSS was slightly higher in the non-ACA diffuse group (27 ± 10) compared with the ACA^+^ diffuse patients (24 ± 10, *P* = 0.075). In addition, ACA^+^ diffuse patients reached peak mRSS later in the disease course (on average, 89 ± 78 months from disease onset) compared with non-ACA diffuse patients (31 ± 33 months, *P* < 0.001).

Patterns of internal organ involvement were different in the three groups (Table 1). Over the entire follow-up period, the ACA^+^ diffuse patients had a higher incidence of ILD (22.9%) compared with ACA^+^ limited patients (4.4%, *P* = 0.001), but lower than non-ACA diffuse patients (41.2%, *P* = 0.042). Likewise, the incidence of SRC was higher in ACA^+^ diffuse subjects (5.7%) compared with none among the ACA^+^ limited ones, but marginally lower than that among non-ACA diffuse subjects (14%, *P* = 0.280). One of the ACA^+^ diffuse patients who had an SRC carried a coexisting anti-RNA polymerase III antibody.

More patients developed PH in the ACA^+^ diffuse group (29%) than in the other two groups (12% in both, *P* = 0.036).

Cardiac involvement was similar in both dcSSc groups (6.2% in the non-ACA diffuse *vs* 8.6% in the ACA^+^ diffuse patients, *P* = 0.484), but much less frequent in ACA^+^ limited patients (1.9% in this group, *P* = 0.074 from comparison with ACA^+^ diffuse).

### Survival analysis

**Fig. 1:**
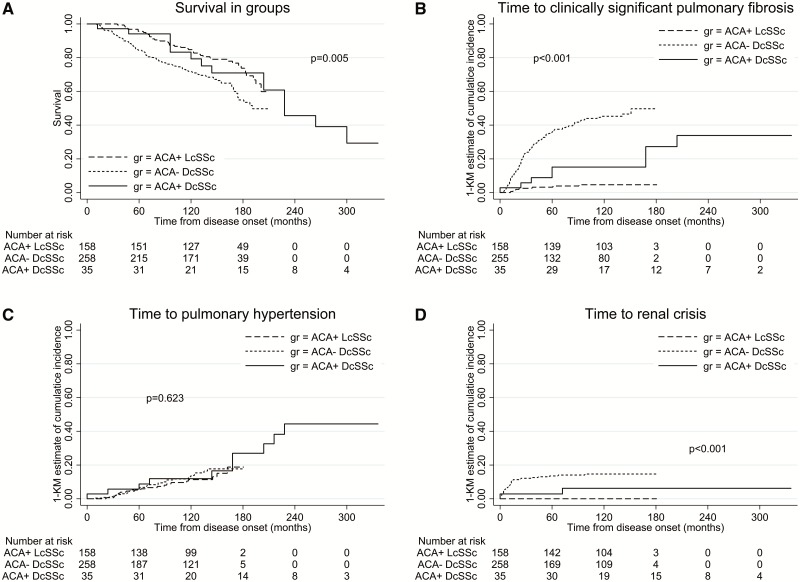
Comparison of survival and cumulative incidence of organ complications between the three groups (**A**) Comparison of survival rate from disease onset between ACA^+^ diffuse, ACA^+^ limited and non-ACA diffuse patients. (**B–D**) Cumulative incidence of interstitial lung disease (**B**), pulmonary hypertension (**C**) and renal crisis (**D**) in ACA^+^ diffuse, ACA^+^ limited and non-ACA diffuse patients. gr: group.

Survival among the ACA^+^ patients was similar for both subsets, with 5-, 10- and 15-year survival rates of 96, 85 and 74% in ACA^+^ limited, and 94, 79 and 71% in ACA^+^ diffuse, respectively (*P* = 0.991). In contrast, non-ACA diffuse patients had much higher mortality, with 5-, 10- and 15-year survival rates of 84, 72 and 55%, respectively, although the difference from ACA^+^ diffuse was not statistically significant (*P* = 0.165; Fig. 1A). As expected, ACA^+^ limited patients had significantly better survival compared with non-ACA diffuse patients (*P* = 0.002).

The incidence of ILD was significantly different between the three groups (*P* < 0.001). During follow-up at 5 years, 15% of ACA^+^ diffuse patients developed ILD, compared with 3% of ACA^+^ limited and 36% of non-ACA diffuse. At 15 years, the cumulative incidence of ILD was 27% in ACA^+^ diffuse, 5% in ACA^+^ limited and 50% in non-ACA diffuse patients (Fig. 1B). The cumulative incidence of ILD in ACA^+^ diffuse patients was sustained over a prolonged period beyond 10 years of the disease course, compared with the non-ACA diffuse group (Fig. 1B).

The cumulative incidence of PH in ACA^+^ diffuse was not different from the other two groups (*P* = 0.621). At 5 years, 9% of ACA^+^ diffuse patients had developed PH, compared with 5% in ACA^+^ limited and 6% in non-ACA diffuse group, and at 15 years the cumulative incidence was 27, 19 and 18%, respectively (Fig. 1C).

The cumulative incidence of SRC was higher in the non-ACA diffuse group (14 and 15% at 5 and 15 years, respectively) compared with the ACA^+^ diffuse (3 and 6% at 5 and 15 years, respectively; *P* = 0.168; Fig. 1D). The cumulative incidence of cardiac involvement was similar in both diffuse groups. At 5 years, 9% of the ACA^+^ diffuse had cardiac involvement compared with 6% of the non-ACA diffuse, and at 15 years it was still 9% in the ACA^+^ diffuse group, whereas it was 7% in non-ACA diffuse. The cumulative incidence of cardiac disease was lower in ACA^+^ limited (1% at 5 years and 3% at 15 years, *P* = 0.065).

In our cohort of ACA^+^ diffuse patients, five harboured autoantibodies that are typically associated with the diffuse subset [antitopoisomerase (ATA), anti-RNA polymerase III (ARA) and anti-U3-RNP antibody; Table 1]. Sensitivity analysis excluding those five patients did not significantly change the results.

Survival rates were not significantly different between ACA^+^ patients with both skin subtypes: 5-, 10- and 15-year survival rates were 96, 84 and 73%, respectively, for ACA^+^ limited and 96, 78 and 73%, respectively, for ACA^+^ diffuse (*P* = 0.86). Non-ACA diffuse also had lower survival rates than ACA^+^ diffuse (84, 72 and 65% at 5, 10 and 15 years, respectively), although it was still not significantly different (*P* = 0.15).

The cumulative incidence of ILD remained different between the three groups (*P* < 0.001). In the ACA^+^ diffuse it reduced when dual antibody patients were excluded to 10% at 15 years, although it remained higher than that in the ACA^+^ limited (5% at 15 years). Likewise, the cumulative incidence of SRC reduced slightly when dual antibody subjects were excluded. At 15 years this was 6% in the whole group of ACA^+^ diffuse patients, and reduced to 4% after exclusion of patients with multiple antibodies. This did not change the overall results of the comparison with non-ACA diffuse and ACA^+^ limited patients (*P* = 0.09). Estimates of the cumulative incidence of PH were also unaffected by exclusion of dual antibody patients, with a cumulative incidence of PH in the ACA^+^ diffuse group not different from the other two groups (*P* = 0.59). At 5, 10 and 15 years of follow-up, this was 9, 12 and 27%, respectively, in the whole ACA^+^ diffuse group and 10, 14 and 26% in the group excluding dual antibodies.

## Discussion

This study confirms that ACA^+^ dcSSc is uncommon and has a distinct clinical phenotype. Among all CTDs SSc is relatively rare and, to our knowledge, there are no studies that specifically describe demographic and clinical characteristics of SSc ACA^+^ diffuse patients.

Similar to our study, the frequency of ACA^+^ diffuse is low in the majority of the SSc registers. In a recent report from the European Scleroderma Trials and Research group (EUSTAR) cohort, 7.2% of the ACA^+^ patients presented the diffuse SSc subset [3]. In the Pittsburgh Scleroderma Database, from a cohort of 1432 patients, 291 were ACA^+^, 5% of them with dcSSc [1]. In another cohort from the German Scleroderma Registry, from 863 patients, ACA was detected in 310 (35.9%) patients, 12 of them (6.9%) with dcSSc [9].

Autoantibodies in SSc are known to be specific and associated with significant clinical manifestations. ACA is one of the hallmark antibodies in scleroderma, targeting centromere protein-B, an alphoid DNA binding protein [2]. ACAs are classically associated with lcSSc, being protective for severe organ involvement, such as cardiac SSc, SRC and ILD [1, 2]. In the Pittsburgh Scleroderma Database, in ACA^+^ patients, 4% had cardiac disease, 1% had SRC and 6% had severe ILD, contrasting with 16% of cardiac disease, 10% of SRC and 23% of ILD in patients with ATA^+^ [1]. Likewise, in the German cohort, patients with ACAs had a lower frequency of ILD [odds ratio (OR) = 0.18 (95% CI 0.12, 0.26), *P* < 0.0001] and cardiac involvement [OR = 0.51 (95% CI 0.32, 0.81), *P* = 0.0033]. Furthermore, ACA^+^ patients were older at disease onset and had more PH [OR = 1.58 (95% CI 0.36, 2.32), *P* < 0.0001] compared with patients carrying other SSc-related antibodies [9]. Our study corroborates these findings, as despite the cutaneous subset, ACA^+^ patients had a lower incidence of both ILD and SRC compared with ACA^−^ patients.

Coexpression of SSc-specific antibodies is rare, although it has been increasingly recognized recently, probably as a result of newer laboratory diagnostic techniques [10, 11]. In our cohort of ACA^+^ diffuse patients, five had dual antibodies that are typically associated with dcSSc. In a EUSTAR group-based study, 0.6% of the patients were double positive for ACA and ATA. In this cohort, double-positive patients had more dcSSc and ILD compared with single-positive patients for ACA, although the incidence of ILD was not significantly different from ATA single-positive patients [10]. Indeed, in a study by Graf *et al*. [11], 14 patients (11%) were positive for multiple SSc-specific autoantibodies, and their clinical phenotype was consistent with the characteristics generally associated with the dominant autoantibody. In the present study, our immunology laboratory reports the dominant patterns to allow unbiased clinical judgement. Nevertheless, sensitivity analysis excluding patients with dual autoantibodies associated with dcSSc did not significantly change the results from the overall group.

In a study by Mierau *et al*. [9] reporting autoantibody specificities and their associations in a German cohort, anti-p25/23 antibodies were identified in a small group of ACA^+^ patients (3.2% of the whole cohort). Clinical characteristics of this subgroup of patients were heterogeneous. Indeed, in that study, subgroup analysis demonstrated that the frequency of ILD was similar between the anti-p25/23 subgroup and the ACA^+^ group as a whole (14 and 13%), with significantly reduced odds for ILD in both groups, compared with patients negative for these antibodies (OR = 0.33 and 0.18, respectively) [9]. However, in a study by Furuta *et al*. [12] ACA^+^ patients with anti-p25/23 antibodies had an increased frequency of ILD. Furthermore, in the study by Mierau *et al.* [9] none of the patients with anti-p25/p23 antibody had diffuse disease, and thus, it is not possible to comment on the frequency of ILD in the ACA^+^ diffuse subset. As this antibody is not available in our laboratory, this association was not evaluated in our cohort.

Disease subset may also influence disease phenotype. Diffuse subset in the EUSTAR database was associated with more internal organ involvement, namely ILD (*P* < 0.001) and SRC (*P* < 0.001), than lcSSc [3]. Indeed, in the present study, ACA^+^ patients with dcSSc had a higher incidence of involvement of internal organs, such as ILD, cardiac and SRC, compared with ACA^+^ limited, despite the known protective role of ACA for these organ complications. However, the involvement of these organs in ACA^+^ diffuse was still less frequent compared with non-ACA diffuse, which might indicate a protective role of ACA in patients with dcSSc.

Analysis of survival in our work seems to reinforce the protective role of ACA in patients with dcSSc. The survival rate in both ACA^+^ subgroups was similar irrespective of the disease subset. Interestingly, although non-ACA diffuse patients had much higher mortality, there was no difference between survival rates in ACA^+^ diffuse and non-ACA diffuse patients. The small number of ACA^+^ diffuse patients might account for these results, or possibly, it might reflect the influence of the diffuse subset in ACA^+^ patients.

In fact, some studies have demonstrated the relevance of dcSSc for early organ involvement, disease severity and survival [13, 14]. Steen *et al*. [13] demonstrated that severe organ involvement in dcSSc often occurs in the first 3 years of disease, and that improvement in skin disease in early dcSSc (<3 years) is associated with better overall survival [5]. Recently, Domsic *et al*. [15] concluded that patients with a rapid skin thickness progression rate had reduced survival [OR = 1.72 (95% CI 1.13, 2.62), *P* = 0.01] and were more likely to develop SRC [OR = 2.05 (95% CI 1.10, 3.85), *P* = 0.02]. In our study, ACA^+^ diffuse patients reached the peak mRSS later than non-ACA diffuse patients and developed clinical significant organ complications, namely ILD, later. Hence, not only does ACA seem to influence survival, but also it can possibly modulate the evolution of organ involvement in dcSSc.

Supporting the clinical heterogeneity of SSc, some authors argue that this is not one defined disease, but a syndrome with different phenotypes. SSc-related clinical characteristics and specific antibodies vary in different countries and ethnicities [16]. Also, familial clustering of the disease, the presence of the same SSc-specific antibodies and HLA class II molecules in families of SSc patients support the evidence that genetic factors contribute to SSc pathogenesis [17]. Several reports and a genome-wide association study in SSc showed its association with HLA class II, IRF5, STAT4 and BANK1 [17, 18]. A strong relationship between HLA haplotypes and specific scleroderma-related autoantibodies is also confirmed, with ACA being associated with HLA class II genes, namely HLA-DQB1 and HLA-DRB1 [18, 19]. These data might reflect immunogenetic heterogeneity in ACA patients and can account for the influence of ACA as a phenotype modifier in dcSSc.

In conclusion, the present study, based on a large single-centre SSc cohort with uniform disease characteristic definitions, confirms that ACA^+^ diffuse is infrequent but has a distinct clinical phenotype. Despite the dcSSc, these patients have a more insidious onset of skin and major organ involvement, which might represent a therapeutic window for early intervention. We confirm that ACA has a protective role and is associated with a lower incidence of ILD and SRC and better survival than expected for dcSSc. ACA, or factors determining its development, may act as a phenotype modifier in dcSSc.
